# Enhancing Immunomodulatory Function of Mesenchymal Stromal Cells by Hydrogel Encapsulation

**DOI:** 10.3390/cells13030210

**Published:** 2024-01-23

**Authors:** Hui-Yun Cheng, Madonna Rica Anggelia, Shiao-Chin Liu, Chih-Fan Lin, Cheng-Hung Lin

**Affiliations:** 1 Center for Vascularized Composite Allotransplantation, Linkou Chang Gung Memorial Hospital, Taoyuan 333, Taiwan; mranggelia@cgmh.org.tw (M.R.A.);; 2 Department of Plastic and Reconstructive Surgery, Linkou Chang Gung Memorial Hospital, Taoyuan 333, Taiwan; 3 School of Medicine, Chang Gung University, Taoyuan 333, Taiwan

**Keywords:** mesenchymal stromal cell, MSC, hydrogel, immunomodulation

## Abstract

Mesenchymal stromal cells (MSCs) showcase remarkable immunoregulatory capabilities in vitro, positioning them as promising candidates for cellular therapeutics. However, the process of administering MSCs and the dynamic in vivo environment may impact the cell–cell and cell–matrix interactions of MSCs, consequently influencing their survival, engraftment, and their immunomodulatory efficacy. Addressing these concerns, hydrogel encapsulation emerges as a promising solution to enhance the therapeutic effectiveness of MSCs in vivo. Hydrogel, a highly flexible crosslinked hydrophilic polymer with a substantial water content, serves as a versatile platform for MSC encapsulation. Demonstrating improved engraftment and heightened immunomodulatory functions in vivo, MSCs encapsulated by hydrogel are at the forefront of advancing therapeutic outcomes. This review delves into current advancements in the field, with a focus on tuning various hydrogel parameters to elucidate mechanistic insights and elevate functional outcomes. Explored parameters encompass hydrogel composition, involving monomer type, functional modification, and co-encapsulation, along with biomechanical and physical properties like stiffness, viscoelasticity, topology, and porosity. The impact of these parameters on MSC behaviors and immunomodulatory functions is examined. Additionally, we discuss potential future research directions, aiming to kindle sustained interest in the exploration of hydrogel-encapsulated MSCs in the realm of immunomodulation.

## 1. Introduction

Advancements in medicine have ushered in the era of cell therapy, applying cellular therapeutics across a broad spectrum of diseases, including degenerative disorders, autoimmune diseases, and certain types of cancer [1]. Mesenchymal stromal cells (MSCs), also known as mesenchymal stem cells, stand out among various cell types due to their remarkable potential in tissue regeneration and immunomodulation.

### 1.1. Potential and Limitations of Clinical Application of MSCs

**Figure 1:**
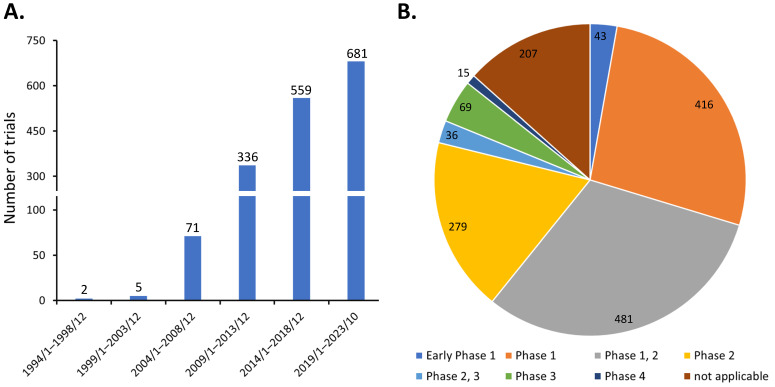
Distribution of MSC clinical trials as of 31 October 2023 by (**A**) initiation date and (**B**) trial phase.

Discovered by Friedenstein et al. around 1970 [2], MSCs are heterogeneous progenitor cells found in various adult and fetal tissues. They display the ability to differentiate into multiple cell lineages, such as osteocytes, chondrocytes, adipocytes, and endothelial cells [3]. MSCs also exhibit potent immunomodulatory effects on various immune cells [4,5,6]. Consequently, MSCs have been investigated in diverse clinical scenarios, as evidenced by the registration of 1654 studies which have been initiated on ClinicalTrials.gov as of 31 October 2023, when the search term ‘(mesenchymal stem cell) OR (mesenchymal stromal cell)’ was queried. The steady growth in the number of clinical trials over the years has demonstrated the great clinical application potential of MSCs (Figure 1A). These efforts have led to the approval of at least ten MSC-based therapies globally, addressing various conditions including myocardial infarction, cartilage degeneration, Chron’s fistula, graft versus host disease, spinal cord injury, and sclerosis [1]. However, the majority of these clinical trials are in phase 1 or 2, with only about 7% (120 of 1654) progressing to phase 3 and 4 (Figure 1B). This suggests that the treatment efficacy of MSCs in many early phase trials may fall short of expectations, thereby limiting their progression to larger-scale studies.

The limitations of MSCs partially stem from their remarkable versatility. The functions of MSCs are influenced by factors such as tissue origin, host physiological/pathological conditions, and the in vitro cell culture environment. For example, adipose tissue-derived MSCs (AD-MSCs) demonstrate a stronger immunosuppressive function than their bone marrow-derived counterparts, BM-MSCs [7]. Disparities in the differentiation capacity and secretome profiles of AD-MSCs emerge in individuals with distinct physiological or pathological conditions, such as cancer or obesity [8]. Moreover, the immunomodulatory capacities of MSCs can be amplified through preconditioning with proinflammatory cytokines like interferon γ (IFN-γ) or tumor necrosis factor α (TNF-α) [9]. However, it has been demonstrated that in vitro cell culture conditions significantly influence the phenotype and functional activities of MSCs. The prolonged culture of MSCs on the polystyrene surface of tissue culture plastics (TCPS) promotes osteogenic differentiation while compromising immunomodulatory potential [10,11]. Concerns arise regarding how in vitro expansion affects in vivo therapeutic efficacy given its necessity in the majority of therapeutic applications [12].

The administration of in vitro-cultured MSCs in vivo presents challenges. Systemic intravenous delivery carries the risk of MSC entrapment in lung vasculature for extended periods, up to 8–9 days [13]. The injection-induced flow stress may also contribute to MSC death. Conversely, locally administered MSCs face the risks of cell washout or death [14]. Consequently, engraftment of the infused cells to the target site typically remains below 5% [15]. Moreover, MSCs may encounter hostile inflammatory environments with activated immune cells at the target site. Therefore, it is crucial to devise strategies to enhance MSC function, alongside improving their survival and engraftment in vivo, in order to fully realize the therapeutic potential of MSCs.

### 1.2. Hydrogel Can Facilitate MSC Application 

One approach to enhance MSCs’ in vivo therapeutic efficacy involves manipulating the cellular environment using biomaterials, such as hydrogels. Hydrogels consist of crosslinked hydrophilic polymers with a high water content [16]. Derived from natural materials like hyaluronic acid (HA), alginate, gelatin (GA), gellan gum (GG), and collagen, natural hydrogels generally exhibit bioactivities, biocompatibility, biodegradability, and low inflammatory and toxicity properties, albeit with week stability and poor mechanical strength [16,17]. In contrast, synthetic hydrogels like polyethylene glycol (PEG), polyvinyl alcohol (PVA), and polyacrylamide (PAAM) offer superior stability and can be engineered with tunable properties [18]. Additionally, semi-synthetic hydrogels, composed of both natural and synthetic polymers, have been developed. In these hydrogels, the synthetic component imparts controlled mechanical properties, while the natural component displays bioactivities [16,19]. Through manipulating the monomer type and concentration, the ratio of crosslinking reagents, and the adjustment of the reaction conditions, hydrogels exhibit versatile properties [16]. Therefore, hydrogels find extensive applications in various biomedical fields, encompassing drug delivery, biosensors, scaffold for tissue engineering, wound dressing, contact lenses, and cell encapsulation [20,21,22]. 

When hydrogels encapsulate MSCs, they provide physical support and protection, creating adhesion sites that mimic cell–extracellular matrix (ECM) interaction [23]. It has been demonstrated that the type of hydrogel impacts MSC lineage differentiation; for example, collagen-encapsulated MSCs tend toward adipogenesis, whereas fibrin leads to poorer adipogenesis but higher angiogenesis [24]. Moreover, the mechanical properties of the hydrogel interact with encapsulated MSCs, influencing their behaviors. For instance, Lee et al. observed that a high cell density in a soft hydrogel helped maintain the multipotency of encapsulated MSCs [25], while a stiffer hydrogel facilitated osteogenic differentiation [26,27]. Encapsulation in hydrogel significantly enhances MSC viability and in vivo cell retention [28,29,30]. Leijs et al. reported that alginate-encapsulated allogeneic MSCs could be retained for over 5 weeks following subcutaneous implantation [31]. The degradation of hydrogel is also a critical issue, particularly for in vivo administration. The mechanisms of hydrogel degradation include biochemical processes such as ester hydrolysis and enzymatic cleavage, environmental factors like pH changes, and user-directed mechanisms such as exposure to light [32,33]. The proper control of degradation ensures that encapsulated cells can exert their function following in vivo administration. For instance, Thai et al. demonstrated that fast hydrogel degradation facilitated embedded MSC migration, the production of angiogenic factors, and wound healing [34]. Hydrogel also provides the flexibility of co-encapsulation of multiple types of cells or with specific drugs or proteins, allowing for further modulation of MSCs within the hydrogel [35,36]. In consequence, hydrogel-encapsulated MSCs have been extensively studied, including in bone regeneration, wound management, and enhancing perfusion following ischemia [37,38,39,40,41]. Clinical studies with promising data have been reported with MSC-laden hydrogels, although not on a large scale (fewer than 40 patients). Examples include HA-encapsulated human umbilical cord MSCs in osteoarthritis patients [42,43,44], as well as human BM-MSCs and AD-MSCs in fibrin sealant on cartilage repair [38]. MSCs have also demonstrated efficacy in treating chronic or diabetic wounds with fibrin spray [45] or direct application with hydrogel on wounds [46,47]. 

Given that immunomodulation is a key feature of MSCs and is critical for their therapeutic function, numerous studies have explored the impacts of hydrogel on MSC immunomodulatory function. In this article, we focus on research that has employed hydrogels to enhance the immunomodulatory effects of MSCs, covering both in vitro and in vivo studies.

## 2. Interactions between MSCs and the Immune System

### 2.1. MSCs on Immune Cells

**Figure 2:**
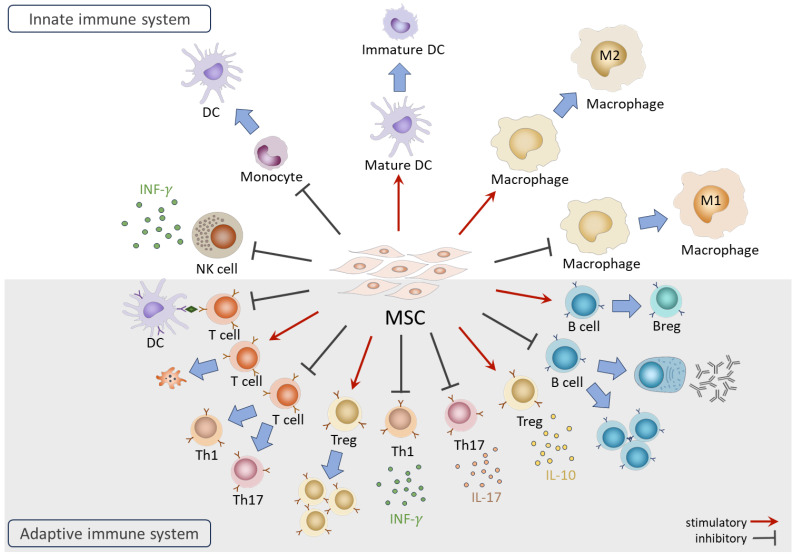
Overview of the functions of MSCs on immune cells.

The immunomodulatory functions of MSCs have been investigated extensively since their discovery. While side-by-side comparisons have indicated that MSC origin plays a role in their immunomodulatory efficacy [48,49], the effects described below on immune cells are common among adult MSCs (such as BM-MSCs and AD-MSCs) and fetal MSCs (including cord blood- and Wharton’s jelly-derived MSCs). Therefore, in this section, that provides an overview of the interactions between MSCs and the immune system, MSC origin is not specified (Figure 2). More detailed information can be acquired in additional reviews [50,51,52]. 

In the innate immune system, coculture with MSCs results in NK cells displaying the suppressed expression of activation receptors, reduced cytotoxic activities, and diminished secretion of IFN-γ [53]. Furthermore, MSCs in coculture exhibit the ability to suppress the differentiation of CD14^+^ monocytes into dendritic cells (DCs) and induce the dedifferentiation of mature DCs into immature form [54]. In the case of monocyte-derived macrophages, MSCs facilitate the generation of anti-inflammatory M2 macrophages while inhibiting differentiation towards the pro-inflammatory M1 subtype [55,56].

In the adaptive immune system, MSCs are renowned for their capacities to suppress effector T cell proliferation and activation through various mechanisms, including arresting the cell cycle of T cells [57], inhibiting antigen (Ag) presentation by DCs [58], inducing T cell apoptosis [59], interfering with differentiation toward pro-inflammatory Th1 and Th17, and promoting the generation of immunomodulatory regulatory T cells (Tregs) [60]. MSCs also impact the secretion of cytokines, suppressing pro-inflammatory IFN-γ and interleukin 17 (IL-17) secretion from Th1 and Th17 cells while enhancing the production of anti-inflammatory interleukin 10 (IL-10) from Tregs [61,62]. For B cells, MSCs influence their activities, inhibiting differentiation, proliferation, and immunoglobulin production [63,64]. Furthermore, MSCs induce the generation of regulatory B cells (Bregs) with the capacity to secrete IL-10 cytokine [5]. 

Many regulatory actions of MSCs on immune cells are mediated by secreted factors such as prostaglandin E2 (PGE2), indoleamine-2, 3-dioxygenase (IDO), human leukocyte antigen-G5 (HLA-G5), TNF-α-stimulated gene/protein 6 (TSG-6), programmed cell death ligand 1 (PD-L1), interleukin 1 receptor antagonist (IL-1RA), and galectin-9 [53,65,66,67,68,69]. Recent studies highlight the involvement of MSC-secreted microRNAs, such as miR-21-5p [70]. On the other hand, cell–cell contact between MSCs and T cells has been shown to contribute to the MSC-mediated suppression of T cell proliferation [71]. It was also reported that the direct transfer of mitochondria from MSCs to Tregs plays a role in maintaining the robust expression of FoxP3 and Treg functionality under inflammatory conditions [72].

### 2.2. Immune Responses on MSCs

MSCs are traditionally considered to be non-immunogenic due to the lack of costimulatory and major histocompatibility complex (MHC) class II molecules [71], and a low level of MHC class I molecules [73]. However, under inflammatory conditions, the expression of both MHC class I and II can be upregulated in MSCs [73,74], potentially eliciting a host immune response that includes the expansion of NK, T, and B cells after in vivo administration [75,76]. A study by Rowland et al. revealed that the repeated intra-articular administration of allogeneic MSCs let to the generation of donor-specific antibodies [77]. These reactions significantly impact the survival and engraftment of MSCs in the host.

## 3. Hydrogel-Enhanced Immunomodulatory Function of MSCs

Hydrogels have been shown to augment the immunomodulatory effects of MSCs in both in vivo and in vitro settings. For instance, Zanotti et al. demonstrated that the subcutaneous administration of alginate-encapsulated BM-MSCs significantly prolonged the survival of mice with graft versus host disease (GvHD) from less than 10 days to over 30 days. In contrast, intravenously administered MSCs did not confer benefits in terms of host survival [78]. In a diabetic wound model, PEG-GA hydrogel-encapsulated human AD-MSCs facilitated wound healing, accompanied by enhanced neovascularization and a significant reduction in macrophage and CD3^+^-T cell infiltration in the wound [79]. Li et al. demonstrated in an in vitro study that collagen hydrogel-encapsulated human umbilical cord (hUC)-MSCs exhibited a more potent attenuation of T cell proliferation than TCPS-cultured MSCs [80]. Moreover, alginate-embedded hAD-MSCs suppressed the proliferation of peripheral blood mononuclear cells (PBMCs) induced by phytohemagglutinin (PHA) and DC maturation [81].

Many hydrogel-embedded MSCs exert immunomodulation by influencing macrophage polarization. This involves a reduction in the proinflammatory M1 population expressing CD86 and inducible nitric oxide synthase (iNOS) and a shift towards an anti-inflammatory and pro-regenerative M2 phenotype (CD163^+^CD206^+^), both in vitro [82] and in animal models [83,84,85]. This mode of action is also observed in MSCs encapsulated by decellularized tissue-derived hydrogel obtained from various tissues such as heart and liver. These hydrogels contain intact ECM, providing favorable microenvironments for embedded MSCs. Studies have demonstrated that these hydrogels can enhance the regenerative potential of MSCs, respond to proinflammatory stimuli, and promote M2 macrophage polarization [86,87].

At the molecular level, hydrogel-encapsulated MSCs exhibit enhanced immunomodulatory function characterized by the attenuated secretion of proinflammatory TNF-α and IL-1β [88,89,90], and augmented production of anti-inflammatory IL-10 and PGE2 [91,92,93]. The expression of the PGE2 production enzyme cyclooxygenase 2 (COX-2) was also studied in parallel, revealing upregulation [80]. Moreover, when the conditioned media (CM) or extracellular vesicles (EVs) of MSCs were collected and encapsulated with hydrogel, improved immunomodulatory function was demonstrated in preclinical wound healing and myocardial infarction models [94,95]. The detailed immunomodulatory mechanisms of these examples are listed in Table 1.

Alternatively, MSCs may induce the production of CD39 and CD73 under inflammatory conditions, leading to the hydrolysis of extracellular ATP and the enhancement of anti-inflammatory adenosine production [96]. Through these molecules, alginate-encapsulated BM-MSCs suppressed dendritic cell maturation, induced Treg differentiation, and prevented the progression of collagen-induced arthritis [97] (Table 1). Additional molecular insights were uncovered through the microarray analysis of hUC-MSCs encapsulated in collagen, chitosan, and PLGA hydrogel. While these three hydrogel types did not exhibit significantly different gene expression patterns from one another, their gene profiles differed significantly from those of TCPS-cultured cells. Many differentially expressed genes, for example *IL-1α*, *IL-1β*, *IL-1RA*, vascular endothelial growth factor (*VEGF*), and hepatocyte growth factor (*HGF*), actively engaged in immunoregulatory networks, influence the immunoregulatory capacities of MSCs [80]. 

In addition to promoting the immunomodulatory functions of embedded MSCs, hydrogel has been identified as a protective shield of MSCs against attacks by the host immune system. For instance, alginate hydrogel shielded encapsulated MSCs from apoptosis induced by cocultured Pan T cells in vitro [98] (Table 1). Silanized hydroxypropyl methylcellulose (Si-HPMC) encapsulation of hAD-MSCs significantly diminished the production of MSC-specific antibodies and lymphocyte-mediated cytotoxicity in a rat irradiation-induced colorectal damage model [99,100] (Table 1). Similarly, Hoban et al. demonstrated that collagen hydrogel-embedded MSCs attracted fewer microglia and astrocytes around the transplantation site, resulting in curtailed immune responses [101] (Table 1). The hydrogel effectively safeguarded MSCs from the chronic inflammatory environment, sustaining the cells in vivo for up to 14 days [79].

**Figure 3:**
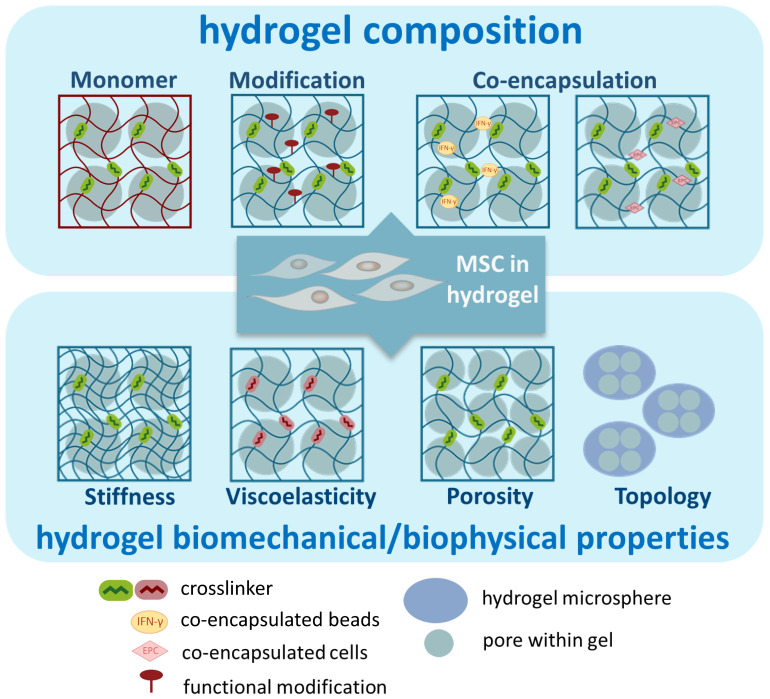
Illustration of parameters of hydrogels to enhance the immunomodulatory functions of MSCs.

The properties of the hydrogel can be controlled by adjusting various parameters, thereby tuning the function of hydrogel-embedded MSCs. Below, we used examples to discuss several parameters that have been investigated to enhance the immunomodulatory functions of embedded MSCs (Figure 3). It is important to highlight that some manipulations may impact multiple properties of the hydrogel. Therefore, the observed improvement in functionality may arise from the interactions between various parameters.

### 3.1. Composition of Hydrogel

#### 3.1.1. The Hydrogel Monomer

Hydrogels display remarkable versatility and slight variations in their composition, resulting in distinct properties that significantly influence the behaviors of embedded MSCs. For example, within the hydrogel composed of high-molecular-weight HA monomers (hHA), hBM-MSCs induced higher IL-10 expression and a greater M2 polarization of monocyte-derived macrophages than their counterparts in the low-molecular-weight HA hydrogel. Notably, hHA counteracted the MSC-mediated suppression of T cell proliferation [102] (Table 1). Kwee et al. reported that MSCs interacted with fibrin and collagen hydrogels through α_v_/α_5_ and α_2_/β_1_ integrins, respectively. In both hydrogels, MSCs with higher integrin levels exhibited the increased suppression of T cell proliferation and higher expression levels of the immunomodulatory IDO1 [103] (Table 1).

Each hydrogel possesses distinct intrinsic properties. For instance, when Wharton’s Jelly-derived MSCs were encapsulated in hydrogel composed of platelet lysate and fibrin, the former demonstrated lower expression levels of *IL-6* and *IL-1β*. This could be attributed to the inherent immunomodulatory properties of the platelet lysate hydrogel, stemming from the rich presence of growth factors and cytokines within, and acting synergistically with MSCs [104] (Table 1). In contrast, MSCs in Si-HPMC hydrogel exhibited higher inducibility against TNF-α and IFN-γ to produce IDO and PGE2 compared to those in alginate hydrogel. The observed differences may also be rooted in the lower stiffness of Si-HPMC hydrogel compared to alginate hydrogel (please refer to the section discussing stiffness below) [105] (Table 1). 

#### 3.1.2. Functional Modification

The C domain of insulin-like growth factor 1 (IGF-1C) serves as the active region of IGF-1, demonstrating pro-survival and mitogenic capacity and actively participating in the healing of tissue injury [106]. Immobilizing IGF-1C in CS hydrogel has been shown to shield encapsulated human placental MSCs from oxidative stress by H_2_O_2_ in vitro and enhance MSC engraftment in vivo. In animal models of acute kidney injury and colitis, IGF-1C-CS encapsulation has proven to enhance MSC therapeutic effects, potentially through an increased secretion of PGE2 and IL-10, the suppression of TNF-α production, and the polarization of macrophages toward the anti-inflammatory M2 phenotype [107,108] (Table 1).

Since hydrogel mimics the function of the ECM for encapsulated MSCs, covalent modification of the hydrogel with the integrin-binding motif, Arg-Gly-Asp (RGD) or Gly-Arg-Gly-Asp-Ser (GRGDS) of fibronectin, can enhance MSC adhesion to the hydrogel. This modification has been shown to improve the survival and reparative functions of embedded MSCs [109] (Table 1), although no significant benefits on immunomodulatory function over non-modified hydrogel have been identified in other studies [81,110,111].

Martin et al. crosslinked a reactive oxygen species (ROS)-degradable poly(thioketal) (PTK) polymer to a PEG-based hydrogel for MSC encapsulation and significantly enhanced MSC viability through protection from ROS-induced cell death. Moreover, the hydrogel was degraded by a ROS-dependent mechanism, enhancing MSC infiltration and MSC-mediated tissue regeneration in an ROS-rich pro-inflammatory environment [112] (Table 1). 

#### 3.1.3. Co-Encapsulation and Pre-Conditioning 

MSCs can be co-encapsulated with other cell types. Zullo et al. encapsulated kidney MSCs with endothelial progenitor cells (EPCs) in HA hydrogel and demonstrated superior therapeutic efficacy against lipopolysaccharide (LPS)-induced endotoxemia compared to MSCs alone in hydrogel. This enhanced efficacy may be associated with augmented M2 macrophage polarization, alteration in macrophage secretome profiles, and the improvement in blood perfusion by EPCs [113] (Table 1). Similarly, the co-encapsulation of insulin-secreting RIN-m cells and MSCs in a hydrogel has demonstrated a synergistic effect in facilitating wound healing. Both cell types exhibited a mutual enhancement in viability and function, showcasing a collaborative therapeutic impact [114] (Table 1).

Preconditioning with pro-inflammatory TNF-α and/or IFN-γ has proven effective in enhancing the immunomodulatory functions of MSCs [9]. Garcia et al. devised a strategy where IFN-γ was tethered to a PEG hydrogel, followed by MSC encapsulation, ensuring in-gel preconditioning and guaranteeing proximity for interaction between IFN-γ and MSCs. This approach significantly augmented the production of IDO and PD-L1, in addition to the suppression of T cell proliferation and DC differentiation [115] (Table 1). The co-encapsulation of IFN-γ-loaded beads with hAD-MSCs in alginate hydrogel also amplified the expression of *IDO* and *galectin-9* [116] (Table 1). In a mouse colitis model, this resulted in boosted PGE2 production and the prolonged induction of galectin-9 expression [117] (Table 1). In a rat myocardial infarction model, the transplanted MSCs maintained immunoprivileged status and were protected from the cytotoxicity of CD8^+^ T cells by the PGE2 being slowly released from the encapsulated hydrogel [118] (Table 1). A reduction in the CD8^+^ T cell population and the consequential protection of encapsulated allogeneic MSCs could also be achieved when the apoptotic Fas ligand (FasL) was co-encapsulated with rat BM-MSCs in an agarose hydrogel [119] (Table 1). The authors demonstrated that the FasL bound to Fas receptor-expressing CD8^+^ T cells to induce apoptosis and had no effects on MSC survival or proliferation. Similarly, co-encapsulation of the anti-inflammatory drug indomethacin suppressed Th1- and Th17-mediated apoptosis of BM-MSC and promoted osteogenesis in a mice bone-defect model [120] (Table 1). Conversely, incorporating a pro-survival and proangiogenic basic fibroblast growth factor (bFGF) in the MSC-laden poly(N-Isopropylacrylamide)-based hydrogel significantly enhanced MSC survival and post-ischemia perfusion in a rat hindlimb ischemia model [121] (Table 1). 

Aiming for a potential therapy for neurodegenerative diseases, Torres-Ortega et al. designed a system where MSCs were co-encapsulated with neurotrophic glial-derived growth factor (GDNF)-loaded nanoparticles in a HA hydrogel. The nanoparticles served to reinforce the HA hydrogel and ensure the slow release of GDNF. The encapsulated MSCs showed an elevated expression of genes in immunomodulatory *IL-4*, *IL-13*, and *IL-10* pathways, as well as the *IL-11* gene [122] (Table 1).

### 3.2. Biomechanical/Physical Properties of Hydrogel

Hydrogel mimics the functions of ECM for the encapsulated MSCs. Therefore, the physical and mechanical attributes of the ECM, such as stiffness, viscoelasticity, topology, and porosity, could play a significant role in influencing cellular behaviors [123]. Research has been conducted to modify these properties of hydrogel with the aim of enhancing the immunomodulatory functions of the encapsulated MSCs.

#### 3.2.1. Stiffness 

The stiffness of hydrogel is influenced by factors such as the extent of crosslinking, the molecular weight, and the concentration of the monomer [124,125] (Table 1). The prolonged culture of MSCs on a stiff surface, such as TCPS, leads to a gradual decline in proliferation, cytokine secretion, and multilineage differentiation capabilities. This decline can be reversed when the MSCs are transferred to culture in PEG hydrogel [126] (Table 1). TCPS have a stiffness at the ~GPa level, in contrast to the ~kPa range found in most tissues [127]. MSCs are sensitive to the mechanical properties of the extracellular environment and employ various pathways to transduce biomechanical signals, including nuclear shuffling of the Yes-associated protein (YAP), Akt kinase, MAP kinases, transcription factors such as RUNX-2, as well as tropomyosin and cytoskeleton machinery [128,129,130]. Therefore, adjusting the stiffness of hydrogel can influence the morphology and secretome profiles of MSCs [124,125]. Ji et al. demonstrated that hBM-MSCs in soft (0.5 kPa) PAAM hydrogel secreted higher levels of paracrine *COX-2*, *TSG-6*, and *IDO* than those in 50 kPa or 200 kPa hydrogel, potentially through regulating actin polymerization. The treatment of F-actin inhibitors to MSCs in stiff hydrogel (200 kPa) significantly increased the secretion of immunomodulatory factors [131] (Table 1). Zhuang et al. encapsulated mice BM-MSCs in 5%, 10%, and 15% gelatin methacryloyl (GelMA) hydrogel, revealing that the soft matrix (5% GelMA) exhibited the strongest immunomodulatory capacity, increasing the production of IL-10 and M2 macrophage polarization. Gene profiling data indicated that immunomodulation was mediated by TSG-6 and the upstream MAP kinase and Hippo pathways [132] (Table 1).

Hydrogel stiffness also contributes to the crosstalk between MSCs and macrophages, affecting the magnitude of the reduction in TNF-α secretion by the M1 macrophage in MSC-macrophage coculture [133] (Table 1). Furthermore, a soft matrix increased the clustering of the TNF receptor 1 (TNFR1) in MSCs, activated NF-kB signaling, and downstream gene expression. This combined effect amplified MSC responses to TNF-α stimulation and enhanced the secretion of paracrine factors. The capability of MSCs to enhance monocyte production in hematopoiesis and monocyte trafficking by C-C motif chemokine ligand 2 (CCL2) under TNF-α stimulation was also enhanced in a soft hydrogel [134] (Table 1).

Although the impact of the extracellular environment’s stiffness on MSC behaviors is acknowledged, different trends exist among studies. For example, Darnell et al. demonstrated that mice BM-MSCs expressed higher levels of *IDO1* and *COX-2* in 18 kPa than 3 kPa alginate hydrogel [135] (Table 1). Other factors, such as cell morphology being spherical or fibroblastic [131], or intracellular signaling pathways influenced by different hydrogels, as described earlier [103], may also potentially interact with stiffness in fine-tuning the immunomodulatory potential of MSCs.

#### 3.2.2. Viscoelasticity

In contrast to stiffness, which reflects how hydrogel responds to static or quasi-static mechanical forces, viscoelasticity describes a more dynamic property with a time-dependent component for the material to respond to the mechanical deformation. Viscoelasticity can be presented by the sum of two components, namely storage modulus (G′) and loss modulus (G″). It has been demonstrated that the types of crosslinkers can influence the viscoelasticity of the hydrogel, and these two components of viscoelasticity may be tuned independently [136]. In an alginate–collagen hydrogel system, viscoelasticity was adjusted by ionic and covalent types of crosslinking. At a fixed G′ of 0.5 kPa and 2.5 kPa, increased viscoelasticity was shown to induce higher levels of *COX-2* and *TSG-6*, while viscoelasticity had no effects on the expression of these two genes in soft (0.25 kPa) hydrogel. The expression of *IL-1RA* was more sensitive to viscoelasticity, suggesting that each gene has different responsiveness to biomechanical cues [137] (Table 1). Morton et al. designed peptido crosslinkers with helical, non-helical, and non-structured secondary structures for a HA-based hydrogel, achieving hydrogels with the storage modulus in the range of 0.6–8 kPa. Although all tested hydrogels endowed embedded MSCs with enhanced expression of immunomodulatory IDO, the softer hydrogel seems to enhance IDO production to a certain degree. IFN-γ supplementation seems to have less effects on softer hydrogels [138] (Table 1).

#### 3.2.3. Topology

Hydrogel may be manufactured in the form of bulk gel or microspheres. The latter possesses a larger surface area and lower barrier that facilitates nutrient and waste diffusion, promoting the viability of encapsulated MSCs after in vivo administration [139]. In contrast to the potentially invasive administration of bulk gel with surgery, microspheres provide the flexibility to be administered intravenously or intra-articularly [140]. Mao et al. employed a microfluidic device to encapsulate MSCs into alginate-derived microgels. When administered intravenously, these microgels exhibited prolonged in vivo retention time and increased the expression of immunomodulatory factors, including *IL-10*, *COX-2*, and *TSG-6*. Furthermore, microgel-encapsulated MSCs facilitated allogeneic engraftment after BM transplantation [141] (Table 1). Rat AD-MSCs encapsulated in gelatin microspheres demonstrated the increased polarization of M2 macrophages, elevated exosome secretion, and enhanced healing of diabetic wounds [142] (Table 1). Geng et al. reported that rBM-MSC-loaded gelatin microcryogels lessened the nephrectomized (NPX)-induced elevation of *TGF-β*, *IL-6*, and *TNF-α* expression and decreased the injury score in the rat chronic kidney disease model [143] (Table 1).

#### 3.2.4. Porosity

Hydrogel is porous material, and a higher porosity of hydrogel, signifying more void volume, generally provides embedded cells with greater capacities to spread, migrate, and engage in intercellular interactions. Qazi et al. increased the average pore size of an alginate hydrogel from 5 nm to 120 µm by freezing and lyophilization without affecting matrix stiffness. MSCs adopted a round morphology within the nano-porous hydrogel; however, a more spread morphology with intercellular connections was observed in the macro-porous gel. Conditioned media collected from the MSCs in macro-porous hydrogel demonstrated enhanced secretion profiles [144] (Table 1). When cultured with an alginate hydrogel with the pore size in the micrometer range, encapsulated MSCs gradually aggregated and formed spheroids within 48 h [97]. MSCs in spheroid expressed higher levels of stemness and immunomodulatory molecules, exhibiting a more suppressive effect on inflammation [145,146,147]. Collagen-encapsulated MSC spheroids demonstrated efficacy in alleviating LPS-induced inflammation. Potential mechanisms included the activation of the PI3K-Akt pathway and increased secretion of immune-related molecules, such as cytokines [148] (Table 1). In a mouse model of irradiation-damaged esophagus, HA-encapsulated MSC spheroids exhibited the ability to suppress proinflammatory TNF-α and IL-1β, concurrently promoting tissue regeneration [149] (Table 1).

**Table 1:** Summary of hydrogel-encapsulated MSCs and their immunomodulatory effects.

MSC ^1^	Hydrogel	Parameter for Comparison	Immunomodulatory Effects	Reference
mMSC	alginate	MSC/hydrogel vs. MSC	(in vivo) lower Ag-induced T cell proliferation in draining lymph node (dLN); prolonged survival of GvHD mice with improved clinical scores	[78]
hAD-MSC	PEG-GA	MSC/hydrogel vs. MSC	(in vivo) prolonged MSC retention; less infiltration of macrophage and T cells; enhanced angiogenesis and post-wound tissue remodeling	[79]
hUC-MSC	collagen, chitosan, PLGA	MSC/hydrogel vs. MSC	(in vitro) all three types increased *IL-1α*, *IL-1β*, *IL-1RA*, *VEGF*, *HGF* expression; collagen hydrogel increased COX-2, IL-1RA, IL-1β protein secretion; suppressed T cell proliferation	[80]
hAD-MSC	alginate	MSC/hydrogel vs. MSC	(in vitro) suppressed DC maturation and PHA-induced PBMC proliferation	[81]
hMSC	HA	MSC/hydrogel vs. MSC	(in vitro) lower CD16 and higher HLA-DR and CD206 expression in cocultured macrophages	[82]
mBM-MSC	n-isopropyl-acrylamide, polyamidoamine	MSC/hydrogel vs. hydrogel	(in vivo) both groups of MSC/hydrogel and hydrogel reduced CD86^+^ M1 macrophage in wound, although no differences in CD163^+^ M2 macrophage were observed; MSC/hydrogel enhanced wound healing with better quality	[83]
neurogenic preconditioned hAD-MSC	GG-HA	MSC/hydrogel vs. control	(in vivo) lower levels of CD163^+^ and CD86^+^ cells; higher M2/M1 ratio in mice diabetic wound	[84]
mBM-MSC	UArg-PEA: GMA-chitosan (ACgel)	MSC/hydrogel vs. MSC or MSC/hydrogel vs. hydrogel	(in vivo) higher cell retention; higher levels of IL-10, M2 macrophage, IL-10-expressing M2 macrophage; lower levels of TNF-α, TNF-α-expressing M1 macrophage	[85]
rAD-MSC	porcine liver ECM	MSC/hydrogel vs. MSC	(in vitro) higher *TSG-6* and *HGF* responding to TNF-α; (in vivo) longer MSC survival; improved pancreatitis	[86]
hUC-MSC	porcine heart ECM	MSC/hydrogel vs. MSC	(in vivo) increased CD206^+^ macrophage; decreased iNOS^+^ macrophage; increased *IL-10*, *IL-13*; decreased *IL-1β*, *TNF-α* in lymph nodes	[87]
GMSC	Nap-GDFDFpDY (pY-Gel) Peptide Hydrogels	MSC/hydrogel vs. MSC	(in vivo) lower levels of IL-1β, TNF-α, and IL-6 proteins; facilitated healing of irradiation-induced wound	[88]
hUC-MSC	GelMA-chitosan-catechol (Chi-C)	MSC/10% hydrogel vs. hydrogel	(in vivo) decreased IL-1β, TNF-α in wound; facilitated wound healing	[89]
hBM-MSC	HA	MSC/hydrogel vs. MSC	(in vitro) lower IL-1β-induced IL-6 secretion; higher IL-1β-induced TGF-β secretion; both groups diminished inflammation of the injured vocal fold	[90]
hBM-MSC	alginate	MSC/hydrogel vs. MSC	(in vitro) lower TNF-α, and higher PGE2 secretion by LPS in asotrocyte coculture	[91]
hBM-MSC	alginate	MSC/hydrogel vs. MSC	(in vitro) higher PGE2/TNF-α ratio induced by LPS; lower LPS-induced IL-1RA, IL-2, IL-6, IL-15, IFN-γ; higher LPS-induced IL-12	[92]
hBM-MSC	collagen	MSC/hydrogel vs. MSC	(in vitro) increased PGE2 secretion and potentiated immunomodulation on macrophages to secrete a higher level of IL-10 and lower level of TNF-α	[93]
pAD-MSC	porcine heart ECM	MSC EV/hydrogel vs. hydrogel	(in vivo) higher CD163^+^CD73^+^, IL-10/TNF-α and lower *CCL-2* in infarction tissue	[94]
hUC-MSC conditioned media	Chitosan/collagen/β-glycerophosphate	MSC-CM/hydrogel vs. unconditioned media/hydrogel	(in vivo) less inflammatory infiltration; enhanced wound healing	[95]
mBM-MSC	alginate	MSC/hydrogel vs. control	(in vivo) extended MSC survival; decreased CD11c^+^, CD86^+^, CD73^+^ and increased Treg in dLN; (in vitro) impairing BM-derived DC maturation through activating adenosine receptor; promoting anti-inflammatory DCs by inhibiting Th1 and Th17 and inducing differentiation of Tregs	[97]
hD-MSC	alginate	crosslinker 200 mM (Alg200) vs. 100 mM (Alg100)	(in vitro) better protection of MSC from Pan-T-induced cell death; (in vivo) less caspase 3 and 8 activity in Alg200-implanted tissue	[98]
hAD-MSC	Si-HPMC	MSC/hydrogel vs. MSC	(in vivo) less MSC-specific antibodies; (in vitro) less *IL-6* expression of M1 macrophage	[99]
rAD-MSC	Si-HPMC	MSC/hydrogel vs. MSC	(in vitro) TNF-α/IL-1β induced higher PGE2, lower TNF-α and IL-8; (in vivo) lower irradiation-induced macrophage recruitment	[100]
rBM-MSC	collagen	MSC/hydrogel vs. MSC	(in vivo) extended MSC retention; lower microgliosis and astrocytosis	[101]
hBM-MSC	HA	HA MWs: high (h, 1.6 M) vs. medium (m, 150 k) vs. low (l, 7.5 kDa)	(in vitro) hHA increased IL-10, decreased IFN-γ and IL-2 production in Th cell coculture; increased CD14^+^CD163^+^CD206^+^ in cocultured monocyte-derived macrophage	[102]
hBM-MSC	fibrin; collagen	fibrin vs. collagen	(in vitro) suppressed CD4^+^ T cell proliferation; increased IDO activity; increased PD-L1 production; different types of integrins were engaged by fibrin versus collagen	[103]
hWJ-MSC	platelet lysate; fibrin	platelet lysate vs. fibrin	(in vitro) lower *IL-6* and *IL-1β* expression when coculture with oxygen-glucose-deprived hippocampal slices	[104]
hAD-MSC	alginate; Si-HPMC	alginate vs. Si-HPMC	(in vitro) lower inducibility against IFN-γ and TNF-α/IFN-γ combination	[105]
hP-MSC	CS-IGF-C; CS	CS-IGF-C vs. CS	(in vivo) better MSC retention and survival; less neutrophil activity; lessened inflammation with lower expression of *IL-1β*, *TNF-α*, *IFN-γ*; higher *IL-10*; higher CD206^+^ and lower iNOS^+^ macrophages	[107]
mAD-MSC	CS-IGF-C; CS	CS-IGF-C vs. CS	(in vivo) enhanced MSC survival; less macrophage recruitment and TNF-α production in acute kidney injury model; accelerate kidney recovery; (in vitro) better protection against H_2_O_2_-induced apoptosis	[108]
rBM-MSC	RGD-hydrogel	MSC/RGD-hydrogel vs. MSC	(in vivo) lower TNF-α, IL-1β, IL-6; longer MSC retention; lower lung injury score (in vitro) higher HGF, VEGF, IL-10	[109]
mMSC	PEG-MAL-PTK; PEG	MSC/PEG-MAL-PTK vs. MSC/PEG	(in vivo) enhanced MSC survival and retention; (in vitro) better protection against H_2_O_2_-induced apoptosis	[112]
mK-MSC	HA	EPC-MSC/hydrogel vs. MSC/hydrogel	(in vitro) higher survival under LPS treatment; (in vivo) higher M2/M1 macrophage following LPS-induced endotoxemia	[113]
hBM-MSC	PEG-diacrylate (PEG-DA)	RIN-m-MSC/hydrogel vs. MSC/hydrogel	(in vivo) produced higher levels of insulin, VEGF, and TGF-β1 and activation of Akt; facilitated wound closure	[114]
hBM-MSC	PEG	MSC/Cys-IFN-γ-PEG hydrogel vs. MSC	(in vitro) increased IDO, PD-L1, CCL2, CCL8 production; suppressed T cell proliferation, DC maturation, (in vivo) better wound repairing	[115]
hBM-MSC	collagen- alginate	MSC in IFN-γ-loaded vs. non-loaded hydrogel	(in vitro) increased expression of *IDO1* and *galectin-9*	[116]
hAD-MSC	alginate	MSC in hydrogel with IFN-γ-beads vs. no beads	(in vitro) higher secretion of PGE2; (in vivo) prolonged secretion of galectin-9	[117]
rMSC	PGE2- hydrogel	MSC/PGE2-hydrogel vs. MSC	(in vivo) enhanced MSC survival and delayed MSC differentiation	[118]
rBM-MSC	FasL- agarose	MSC/FasL-agarose vs. MSC/agarose	(in vivo) MSC retention; reduced CD8^+^ T cell population at injury site; increased secretion of IL-1RA	[119]
hBM-MSC	RGD-alginate; poly ethylene glycol dimethacrylate (PEGDMA)	MSC in nanoporous (alginate, PEGDMA) vs. microporous hydrogel	(in vivo) less infiltration of TNF-α, IL-17 cytokines and Th17; lower caspase 3/8 activities; higher survival of MSC	[120]
rBM-MSC	bFGF-N-isopropylpolyacrylamide	MSC/bFGF-hydrogel vs. MSC/hydrogel	(in vivo) enhanced MSC retention and survival	[121]
rBM-MSC	GDNF-HA	MSC/GDNF-hydrogel vs. MSC/hydrogel	(in vitro) RNA profiling showed enhanced *IL-4*, *IL-10*, and *IL-13* signaling, and *IL-11* gene; enrichment of anti-inflammatory suppressor of cytokine signaling (*SOCS2*)	[122]
hBM-MSC	HA-GA	MSC in hard (20 kPa) vs. soft (2 kPa) hydrogel	(in vitro) different stiffness of hydrogel elicited different secretome profile	[124]
hBM-MSC	PEG-DA	MSC in soft (30 kPa) vs. hard (100 kPa) hydrogel	(in vitro) an overall increase in abundance of immunomodulatory factor secretion. Each cytokine has unique elasticity-dependent response pattern	[125]
hBM-MSC	PEG	MSC/hydrogel vs. MSC	(in vitro) hydrogel rescue MSC phenotype change post expansion on TCPS; enhanced cytokine secretion	[126]
hMSC	PAAM	MSC in soft (0.5 kPa) vs. medium (50 kPa) vs. rigid (200 kPa) hydrogel	(in vitro) enhanced PGE2 secretion; *COX-2*, *TSG-6*, *IDO*, *IGF-1* expression; CM polarizes M2 macrophage; inhibition of actin polymerization rescued the secretion profiles of MSCs cultured on 200 kPa hydrogel	[131]
rBM-MSC	GelMA	MSC in soft (5% GelMA) vs. medium (10%) vs. stiff (15%) hydrogel	(in vitro) induced lowest intensity of iNOS; lowest TNF-α, IL-1β, and highest IL-10 secretion in macrophage coculture; (in vivo) induced lowest M1 macrophage and highest M2 macrophage	[132]
hBM-MSC	collagen-coated PAAM	MSC in soft (11 kPa) vs. medium (88 kPa) vs. rigid (323 kPa) hydrogel	(in vitro) medium hydrogel showed the largest suppression of TNF-α secretion and enhancement of IL-10 production in macrophage-MSC coculture	[133]
hBM-MSC	alginate	MSC in soft vs. stiff hydrogel	(in vitro) higher expression of *CCL-2*, *IL-6*, *IL-8*, *TSG-6* in MSCs by TNF-α treatment; larger cluster of TNFR1 on MSC; (in vivo) promotes the MSCs to produce and recruit monocytes upon TNF-α stimulation	[134]
mMSC	alginate	MSC in soft (3 kPa) vs. medium (18 kPa) vs. rigid (30 kPa) hydrogel	(in vitro) higher expression levels of *IDO1* and *COX-2* in 18 kPa than 3 kPa hydrogel. *COX-2* expression is sensitive to TNF-α/IFN-γ stimulation in soft matrix	[135]
hBM-MSC	alginate- collagen	MSC in hydrogel with stiffness (0.25–3 kPa); viscoelasticity (by loss angle 2–8)	(in vitro) gene expression of *COX-2* and *TSG-6*, and PGE2 secretion, were regulated by both stiffness and viscoelasticity. *IL-1RA* was more sensitive to viscoelasticity	[137]
hMSC	HA	MSC in hydrogel with crosslinker: helical vs. non-helical vs. unstructured; and peptide length 14 vs. 8	(in vitro) all hydrogels increased IDO secretion; softer hydrogel seems to enhance IDO production to certain degree. The IFN-γ supplementation had less effects on softer hydrogels	[138]
mMSC	alginate	MSC/microgel vs. MSC	(in vivo) increased MSC retention time; increased *IL-10*, *COX-2*, *TSG-6* gene expression; enhanced allogeneic bone marrow cell engraftment	[141]
rAD-MSC	GA	MSC/GA microsphere vs. GA microsphere	(in vivo) enhanced M2 macrophage polarization, exosome secretion, and wound closure in diabetic rats	[142]
rBM-MSC	GA microcryogel (GM)	MSC/GM/NPX vs. NPX	(in vivo) suppressed NPX-induced *TGF-β*, *IL-6*, *TNF-α* expression	[143]
rAD-MSC	alginate	MSC in microporous vs. nanoporous hydrogel	(in vitro) enhanced overall secretome profile, including IGF, VEGF, and HGF	[144]
rAD-MSC	collagen	MSC spheroid size in hydrogel vs. hydrogel	(in vitro) in the neural stem cell coculture system, suppressed LPS-induced secretion of TNF-α and PGE2 with spheroid size-dependent activity; secreting higher levels and types of cytokines and immune-related molecules	[148]
hAD-MSC	catechol modified HA	MSC spheroid/hydrogel vs. MSC spheroid	(in vivo) extended MSC survival; decreased *TNF-α*, *TGF-β*, *IL-1β* gene expression	[149]

^1^ h, m, and r stand for the MSCs isolated from human, mice, and rat origin. AD, BM, G, UC, WJ, D, K, and P stand for the tissue sources being adipose tissue, bone marrow, gingival, umbilical cord, Wharton’s Jelly, dental, kidney, and placenta.

## 4. Perspectives and Future Directions

The integration of hydrogel encapsulation into MSCs presents a promising avenue for advancing immunomodulatory functions. To optimize this strategy, a deeper understanding of the mechanistic insights into MSC-hydrogel interactions is essential. Innovative technologies such as 3D printing have leveraged physiological knowledge to produce tailored hydrogel/scaffold for diverse application. For instance, the weight-bearing property of administered hydrogel is crucial for addressing bone defects or cartilage injury [150]. The property of injectability may be more critical for intraarticular or intracardiac applications than wound management. Therefore, the ideal combination of hydrogel and MSC depends on the physiological condition it aims at. 

On the other hand, as our understanding of the interactions between hydrogel and other immune cells grows [151], designing hydrogels with intrinsic immunomodulatory properties for specific clinical settings that synergize with MSCs offers potential benefits. For example, the n-isopropylacrylamide (NIPAM) hydrogel independently reduced the expression of M1-macrophage markers in a mouse diabetic ulcer model and enhanced the efficacy of MSCs [83]. Incorporating immune-regulating drugs is another viable approach, as demonstrated in the co-encapsulation of BM-MSCs with the immunosuppressant cyclosporin A in a PLA scaffold to the mice with allogeneic skin grafts. The scaffold demonstrated reduced *IL-1β*, *IFN-γ* expression, and increased M2 macrophage polarization within the graft [152]. The flexibility of hydrogels to encapsulate both drugs and cells allows for engineering systems that release drugs modulating MSC function under specific environment cues, such as a change in pH or temperature. This approach holds promise for other immune-related conditions, including organ or tissue allotransplantation. MSCs have shown potential to induce donor-specific tolerance in allotransplantation settings [153,154], and future studies incorporating hydrogel and MSCs may alleviate patients from the burden of life-long immunosuppressants. 

However, transition from in vitro to in vivo environments introduces additional physiological complexities. Certain hydrogel types may trigger a foreign body response in vivo, leading to inflammation responses, fibrosis, and encapsulation by cells and collagens [155]. In-depth investigations to monitor the administered MSC-hydrogel complex post-administration are crucial for understanding long-term efficacy and safety, guiding adjustments to dosage and frequency, and enhancing therapeutic outcomes.

Despite its great promise, hydrogels do have limitations. For example, HA has low cell adhesive capacity, and alginate exhibits low biodegradability and a potential immunogenic response. Synthetic hydrogels like PEG are biologically inert with a limited cell adhesive capacity, high costs, and fewer in vivo studies [156]. Although more amenable for modification, careful consideration of the in vivo degradation of synthetic hydrogel is necessary to prevent potential issues. The degradation rate is another challenge; ideally, hydrogels should degrade at a rate that allows tissue integration and cell function without leaving behind harmful byproducts. Faster degradation rates have been associated with decreased cell viability [157]. On the manufacturing side, natural and synthetic hydrogels each have challenges, such as batch-to-batch variations and difficulties in scaling up, respectively.

The current data suggest the potential to enhance the immunomodulatory function of MSCs through hydrogel encapsulation, offering a promising strategy for improving the therapeutic efficacy of MSC. Moving forward, the necessity for more well-controlled clinical trials has become evident, as they are essential for evaluating the safety and efficacy of MSCs in hydrogels for specific medical conditions, and translating these promises into tangible therapeutic advancements. 

## 5. Conclusions

While the immunomodulatory capacities of MSCs have long been recognized, realizing their full potential as cell therapeutics in vivo has been impeded by challenges like the inflammatory environment and limited engraftment. The encapsulation of MSCs in various hydrogels has emerged as a promising strategy, demonstrating the potential to enhance their immunomodulatory function in vivo. Notably, hydrogel parameters, including compositions like monomer type, functional modification, and co-encapsulation, along with biomechanical and physical properties such as stiffness, viscoelasticity, topology, and porosity, have been shown to finely tune the immunomodulatory functions of the encapsulated MSCs. The next crucial steps involve designing hydrogels tailored for encapsulating MSCs under specific conditions and conducting well-controlled clinical studies to validate the therapeutic potential of this approach.

## Data Availability

This is a narrative review based on published data.
